# Profiles of Quality in Three Distinct Early Childhood Programs Using the Brief Early Childhood Quality Inventory (BEQI)

**DOI:** 10.1007/s13158-022-00344-9

**Published:** 2023-01-14

**Authors:** Abbie Raikes, Rebecca Sayre Mojgani, Jem Heinzel-Nelson Alvarenga Lima, Dawn Davis, Cecelia Cassell, Marcus Waldman, Elsa Escalante

**Affiliations:** 1 College of Public Health and ECD Measure, University of Nebraska Medical Center, Omaha, USA; 2 ECD Measure, Omaha, USA; 3 University of Nebraska-Lincoln, Lincoln, USA; 4 University of Liberia, Monrovia, Liberia; 5 Universidad del Norte, Barranquilla, Colombia

**Keywords:** Early childhood care and education, Quality measurement, Cross-cultural child development, BEQI

## Abstract

Quality early childhood care and education (ECCE) is important for young children’s holistic healthy development. As ECCE scales, contextually relevant and feasible measurement is needed to inform policy and programs on strengths and areas for improvement. However, few measures have been designed for use across diverse contexts. Drawing on principles of mixed methods design, this study reports on a new approach to ECCE quality measurement: the Brief Early Childhood Quality Inventory. Using data from the USA, Liberia, and Colombia, results indicate variation in the items perceived as highly relevant to each setting and in the characteristics of classrooms including the degree of child autonomy, the types of activities, and in child/educator interactions and dialogue. However, despite this variation, a small set of items indicate potential functionality as cross-country anchor items. Findings lend support to the idea that quality measures can have some common elements with room for adaptation within and across settings. Future work in this area should address the possibility that the significance of these practices for child development also varies across settings.

## Résumé

La qualité de l'Education et de la Protection de la Petite Enfance (EPPE) est importante pour le développement sain et holistique des jeunes enfants. À mesure que l’EPPE évolue, des mesures contextuellement pertinentes et réalisables sont nécessaires pour informer les politiques et les programmes sur les points forts et les domaines à améliorer. Cependant, peu de mesures ont été conçues pour être utilisées dans divers contextes géographiques. S'appuyant sur les principes des méthodes mixtes, cette étude présente une nouvelle approche de la mesure de la qualité de l’EPPE appelé le Bref Inventaire de la Qualité de la Petite Enfance. À l'aide de données provenant des États-Unis, du Libéria et de la Colombie, les résultats indiquent des variations dans les éléments perçus comme très pertinents dans chaque contexte et dans les caractéristiques des salles de classe, y compris le degré d'autonomie de l'enfant, les types d'activités et les interactions et le dialogue entre l’enfant et l’éducateur. Cependant, malgré cette variation, un petit ensemble d'éléments indique une fonctionnalité potentielle en tant qu'éléments d'ancrage transnationaux. Les résultats appuient l'idée que les mesures de la qualité peuvent avoir des éléments communs avec une marge d'adaptation dans et entre les localités. Les travaux futurs dans ce domaine devraient aborder la possibilité que l'importance de ces pratiques pour le développement de l'enfant varie également d'un endroit à l'autre.

## Resumen

La calidad de la atención y la educación de la primera infancia (AEPI) es importante para el desarrollo holístico y saludable de los niños pequeños. A medida que la AEPI escala, se necesita una medición contextualmente relevante y factible para informar las políticas y los programas sobre las fortalezas y las áreas de mejora. Sin embargo, pocas medidas han sido deseñadas para el uso entre contextos diversos. Basándose en los principios del diseño de métodos mixtos, este estudio informa sobre un nuevo enfoque para la medición de la calidad de la AEPI: el Inventario Breve de la Calidad de la Educación Inicial. Utilizando datos de los EE.UU, Liberia, y Colombia, los resultados indican una variación en los ítems percibidos como muy relevantes para cada entorno y en las características de las aulas, incluido el grado de autonomía del niño, los tipos de actividades, y en las interacciones y el diálogo entre los niños y los educadores. Sin embargo, a pesar de esta variación, un pequeño conjunto de ítems indica una funcionalidad potencial como ítems ancla entre países. Los hallazgos apoyan la idea de que las medidas de calidad pueden tener algunos elementos comunes con espacio para la adaptación dentro y entre entornos. El trabajo futuro en esta área debería abordar la posibilidad de que la importancia de estas prácticas para el desarrollo infantil también varie según los entornos.

## Introduction

Substantial empirical and theoretical work affirms the importance of young children’s experiences for their holistic long-term health, learning, and well-being (Black et al., 2017; Engle et al., 2011; Shonkoff & Phillips, 2000). Countries around the world are investing in early childhood care and education (ECCE) programs (Richter et al., 2017). In this paper, we refer to ECCE as encompassing programs designed to support both care and education as critical to young children’s development (Langford et al., 2017). In many countries, a large proportion of parents are employed, leading to a reliance on early childhood settings for care as well as education (International Finance Corporation, 2017). Early childhood settings can include several types of programs, from formal, school-based preschool settings to community- and home-based childcare, ranging from a few hours a week to forty hours a week or more and serving children from birth through the start of school with a focus on attending to children’s needs for both care and education (Author citation redacted).

As access to ECCE expands, a key question is whether the quality is high enough to ensure children’s healthy development and learning (Marope & Kaga, 2015; Yoshikawa et al., 2018); in particular, high enough quality to address inequities in early childhood due to inadequate health care, nutrition, and social protection among other factors (Britto et al., 2017). One approach to improving quality is to routinely monitor all early childhood settings to index quality practices (Gullo, 2013; Yoshikawa et al., 2018). Data on quality of ECCE settings can play a powerful role in influencing early childhood systems, by providing feedback to policymakers and ECCE professionals on overall levels of quality and places to invest in quality improvement (Thorpe et al., 2022; Zaslow et al., 2011). Generating useful data on early childhood systems requires measures that are valid and reliable, as well as contextually relevant and feasible to use at scale. While indicators of access to ECCE such as enrollment in preprimary education are routinely collected across countries (UNESCO, 2016), there are no agreed upon global indicators of quality or recommendations for quality measurement tools, leading to a lack of information about children’s learning and development in many parts of the world (Author citation redacted). This study reports on a new approach to quality measurement, called the Brief Early Childhood Quality Inventory (BEQI), designed to provide contextually relevant information on quality across countries using a simple, adaptable tool.

### Defining Quality Across Settings

Quality in early childhood settings has been defined based on both theory and empirical evidence. Key aspects of quality include: well-trained and compensated early childhood professionals (Torquati et al., 2007; Manning, 2019); supportive, stimulating interactions between educator and children (Ulferts et al., 2019); children’s access to developmentally appropriate toys and materials (Mashburn et al., 2008); focused attention on literacy and math (Jenkins et al., 2018); children’s ability to play and drive their own learning (Zosh et al., 2017); and safe, healthy environments (Organization for Economic Cooperation & Development [OECD], 2012). Quality in early childhood programs has been conceptualized as consisting of structural quality (e.g., the characteristics of educators and settings such as access to training and education levels) and process quality (e.g., the quality of interactions and activities that children experience while they are attending an ECCE setting) (NICHD Early Child Care Research Network, 2002; Burchinal, 2018). Effects of structural quality are thought to be mediated through process quality: structural quality (e.g., educator training or ratio requirements) influences process quality (e.g., early childhood professionals’ interactions with children), which in turn affects child outcomes (Mashburn, 2008).

Although empirical and theoretical work outlines key aspects of quality, there is little work documenting quality in diverse early childhood settings (Chen & Wolf, 2021). Some work has raised the question of whether cross-cultural definitions of quality are appropriate or desirable, given the diversity in child-rearing values and goals around the world (Dahlberg et al., 1999; Myers, 2004). Further, country income and investment lead to substantial impacts on education systems (Hossain & Hickey, 2019) including ECCE settings. In countries with less income, this is manifested through lack of access to professional development (Schwartz et al., 2019; Sun et al., 2015), under-resourced classrooms (Raikes et al., 2020), and low levels of access to stimulating home and out-of-home learning environments (McCoy et al., 2018), and thus is another source of variation. Despite variation in quality due to culture and country income, theoretical and empirical work on quality and child development suggests that some aspects of children’s environments should have consistent impacts regardless of settings. For example, exposure to emotionally supportive and cognitively stimulating environments promotes learning and development in all settings (Britto et al., 2017) and positive impacts of ECCE on children’s development have been documented in studies conducted around the world (Rao et al., 2017; von Suchodoletz, under review). However, less work to date has delineated specific elements of ECCE environments that may either have consistent impacts or vary based on context, as we outline in greater detail below.

Below we outline two aspects of process quality that may vary based on cultural and contextual influences as an example of central aspects of children’s environments that may vary based on context. These two dimensions were selected based on existing literature that identifies their role in promoting children’s development as well as the potential impacts of culture and context in influencing their manifestation. While impactful for children’s development, these two dimensions should not be considered indicative of the relative importance of these dimensions over others (for example, the quality of child/educator interactions, which has been extensively documented as an important component of ECCE quality) (Bergin et al., 2009; Mashburn, et al., 2008).

#### Child Choice Vs. Early Childhood Professional-Directedness

A high degree of child choice in early childhood classrooms is a feature of autonomy support, or the extent to which children are supported in following their own interests and leading their own learning. Child choice can be observed by children choosing their own materials, having open/free play time, or given options to choose what they want to do. Autonomy support has been linked to several positive aspects of child development, including increased self-regulation (Cadima et al., 2019). However, dimensions of autonomy and control are culturally embedded, and thus these dimensions must be understood within the larger context of societal goals for child-rearing which in turn influence how children understand and experience autonomy and control (Serpell, 2011; Soenens et al., 2015; Tobin, 2005). Although some country settings emphasize autonomy in early childhood settings, others prioritize interdependence and interconnection (Tobin, 2005). While global emphasis has recently shifted toward a definition of quality that prioritizes child directedness in ECCE, it is unclear how this idea resonates with prevailing norms and expectations for child development in many parts of the world (McCoy, 2022).

A related construct is the amount of time children spend in whole group vs. individual activities in ECCE. In the USA, time spent in structured activities has been associated with greater gains in school readiness, while time spent in free play has been associated with gains in self-regulation (Goble & Pianta., 2017) and greater child engagement (Hooper et al., 2017), with more developmental gains for children whose educators provided some degree of learning support during free play (Goble & Pianta., 2017). Intentional time spent on learning activities in ECCE, particularly literacy, mathematics, and science, leads to greater learning gains at the start of primary school (Burchinal, 2018). However, in some countries, there is a high priority placed on academic preparation in early education, which can translate into more direct instruction than free play (e.g., in India (Sriprakash et al., 2020) and in China (Li et al., 2014)). Whole group instruction is common in many early childhood settings, even when there is emphasis on increasing time spent in small group activities. Features of effective whole group settings that encourage children’s attention have been identified such as: ensuring that all children have materials; materials are used; early childhood professionals respond to children’s communications; and early childhood professionals relate the whole group activity to children’s experiences (DiCarlo et al., 2012).

#### Language Environments

Early childhood language environments are critical to long-term development. Children who hear more words and who participate in more exchanges with adults in the early childhood years show increases in cognition later in childhood and in adolescence (Gilkerson et al., 2018; Huttenlocher et al., 2010). The quality of inputs to children, as well as the quantity, is predictive of children’s later development (Anderson et al., 2021; Rowe & Snow, 2020). Quality of discourse includes several components, including the extent to which discourse is interactionally supportive, linguistically adapted, and conceptually challenging for children (Rowe & Weisleder, 2020). Interactionally supportive dialogue captures children’s attention and responds to children’s interests and encourages neurological development (Cartmill, 2016; Romeo et al., 2018). Linguistically adapted dialogue refers to the level of complexity in words and grammar (Lieven et al., 2019), while conceptually challenging refers to dialogue that meets children’s conceptual needs by introducing ideas that help children stretch to a new level of understanding (e.g., referring to past, present, or future in conversations with very young children) (Rowe & Snow, 2020). Yet as with other aspects of quality, there is presently little research that outlines characteristics of dialogue within early childhood settings in different ECCE settings. A strong body of work has identified the socio-cultural influence on language and discourse between parents and young children, from the distinct types of words parents use in talking with children to the complexity of sentences and response patterns (Nelson, 1998; Nelson et al., 2004). This work has identified notable differences in how children are spoken to in different contexts, with much less child-directed speech typical in some contexts compared to others, especially in less developed countries Cristia et al., 2019; Weber et al., 2017). Less work has described language interactions in out-of-home settings across contexts. Given the theoretical and empirical work addressing cultural influences on language interactions, language environments may vary quite considerably from one context to the next.

### Existing Measures of ECCE Quality

A small set of tools has often been used to measure quality in early childhood settings: the Classroom Assessment Scoring System (CLASS) (Pianta et al., 2008) and the Early Childhood Environmental Ratings Scales (ECERS) (Harms et al., 1998). Chen and Wolf, (2021) offer a brief review of measurement specifically within low- and middle-income countries and Burchinal (2018) outlines commentary on use of existing scales within high-income countries. Although these measures were originally designed for high-income countries, adaptations are often used in low- and middle-income countries (Fernald et al., 2017). Two recently developed tools, the Measuring Early Learning Environments Scale (MELE) (UNESCO, 2017) and the Teacher Instructional Practices and Processes System (TIPPS) (Seidman et al., 2014), were designed specifically for low- and middle-income country settings and have been used in several countries (Chen & Wolf, 2021; Ponguta et al., 2020; Raikes et al., 2020). Across all scales, there is an emphasis on capturing professional/child interactions, and the extent to which early childhood professionals scaffold children’s learning; engage in rich, stimulating discussions; and meet the developmental needs of children in ECCE settings.

A central premise of quality measurement is that children who attend higher-quality early childhood settings as measured by these tools will show improved developmental outcomes (Burchinal, 2018), underscoring the importance of quality for promoting equity in early childhood. The positive impacts of high-quality ECCE on child development have been documented using both randomized and associative study designs, although effect sizes tend to be small. In high-income countries, research has demonstrated small to moderate associations between quality and child outcomes using the ECERS (Brunsek et al., 2017) and the CLASS (Perlman et al., 2016). In low- and middle-income countries, there is less evidence on the size of associations between scores from quality measures and child development, although a growing body of work has indicated associations between higher ECCE quality and child development, for example: in Ghana using the TIPPS (Wolf & McCoy, 2019); in one sub-Saharan country using MELE (Raikes et al., 2020); in Chile using MELE (Leyva et al., 2015); and in Colombia using MELE (Maldonado-Carreño et al., 2018). Taken together, the small effect sizes suggest two conclusions: first, that the mix of items and constructs in commonly used quality measures may be only one way of defining and indexing quality in early childhood settings; and second, that additional work to describe early childhood settings may be useful in identifying patterns and profiles across contexts that in turn can be used to inform contextually relevant tools.

Beyond small associations with measures of child development, research using existing quality measures has revealed several measurement challenges (Burchinal, 2018; Gordon et al., 2021). One challenge is the limited empirical evidence documenting that scales function in similar ways across countries and that low scores on scales have the same meaning in each context (Betancur et al., 2021). Second, when importing definitions of quality across contexts, it is not clear that a low score truly indicates lower levels of quality, especially in the absence of full-scale validation in each new context in which it is used (Thorpe et al., 2022). As a result, there is a tendency for floor effects to emerge in some settings, especially in low- and middle-income countries but also in some settings in high-income countries that represent diverse settings and/or settings that are new to quality measurement (Betancur et al., 2021; Thorpe et al., 2022). Third, the complexity of existing scales leads to difficulty in training observers to reliability standards and interpreting scores (Burchinal, 2018; Raikes et al., 2020), a key step in ensuring that data are used to improve early childhood settings. Training requirements can exacerbate resource constraints when using measures at scale, as observers need substantial training to understand and apply the concepts embedded in tools (Raikes et al., 2020).

### Using Data For Improvement

Defining how measures can be designed and implemented to help improve ECCE settings is important for ensuring quality for all children and particularly for using ECCE settings to promote equity, especially given the limited resources available to invest in quality in many countries. Both the content and the structure of existing scales add to the challenges in using data for improvement. In primary schools, early childhood professionals’ perceptions of the relevance of the research to their own settings influences their willingness to adopt evidence-based practices (Joram et al., 2020). When early childhood professionals perceive a disconnect between research findings and their own settings or when specific changes to make are hard to identify, willingness to adopt new practices may be limited. Wolf et al. (2022) report that early childhood professionals in Ghana do not view all practices within the CLASS scale as relevant to their contexts. Davis et al., (2021) found that there was agreement on key aspects of quality as measured in the MELE, but little support to implement quality practices. Adaptations of MELE have revealed that many countries require additions or changes to quality scales before considering them appropriate for the setting (Raikes et al., 2020). Even in countries where quality measures have been developed, specific groups of early childhood professionals report feeling that the measures do not represent their practices well, leading to overall skepticism of quality measurement (Tonyan et al., 2017). Results from existing measures can be difficult to translate into practice, in part because existing scales’ structures make it challenging to identify specific changes to make (Hanno et al., 2021). The perceived lack of alignment and inability to implement changes may lead to limited impact of the data from quality measures on changes in practices. In sum, measurement is difficult to scale and even more difficult to interpret and apply to quality improvement, which contributes to limited insight into quality practices within many settings and risks generating misleading or inaccurate estimates of ECCE quality.

The purpose of this study is to respond to the stated need for new approaches to cross-contextual quality measurement (Chen & Wolf, 2021) by providing descriptive information on a set of evidence-based practices in early childhood educational settings in three distinct country contexts: the USA (high income), Liberia (low income), and Colombia (upper middle income) (World Bank, 2021). The study design employed draws on principles of mixed-method research by highlighting the need to understand contextual influences on ECCE quality especially in real-life ECCE settings (Creswell et al., 2011). This study was conceptualized as a first step in documenting differences across settings, which in turn can lay the groundwork for more extensive analyses on cultural and contextual influences on quality. We pose two main research questions: 1) what are the characteristics of each of these three types of early childhood settings, using a checklist of quality indicators from previous research rather than one of the existing measures of ECCE quality?; and 2) which, if any, of these indicators show initial signs of applicability across countries? Conclusions from this study are intended to provide insight into approaches that could be used to create contextually relevant and feasible measures of quality across contexts.

Because we were interested in looking at highly diverse sites, the sites vary considerably in the number and age of children in each classroom, educator qualifications, and regulatory contexts. While the countries represented are diverse in terms of population and income, the countries were selected based on existing research partnerships and the expertise and interest of lead researchers in each setting. We selected family childcare in the USA as the type of care to focus on because of the identified need to define quality in family childcare based on the cultural and contextual features that are different from center-based settings (Tonyan, 2017). Settings in Colombia and Liberia were selected based on the desire to compare practices across high, middle, and low-income country settings given the potential impacts of country income on typical ECCE settings. All sites were identified in collaboration with researchers within each country, based on geographic proximity and feasibility of collecting data. Below we provide a short description of the early childhood programs that are included in our study.

#### USA

In the USA, family childcare providers are individual proprietors who care for children in their homes, with state licensure required if providers are paid to care for more than three children who are not family members. Children range in age from birth through start of school, and most states require that family childcare providers are licensed for basic health and safety practices and regulated through an annual licensing visit. In Nebraska, where these data were collected, licensing standards do not include any specification of learning environments and instead are only focused on health and safety standards, although the state also has quality standards as outlined in early learning guidelines (State of Nebraska, n.d.). There is a state-run voluntary quality improvement system with no publicly available information on how many family childcare providers take part in the system. A limited body of work has examined the quality of family childcare settings. Overall, research on family childcare has focused more on physical environments than on learning environments (Bromer et al., 2021). Findings indicate that family childcare has characteristics that differentiate it from center-based care, including greater attention to the social/emotional development of young children and less attention to preparing children for school through structured activities and use of a curriculum (Bromer et al., 2021). Both family childcare providers and researchers have articulated gaps between positive aspects of family childcare programs and existing quality measures, such as longevity in bonds between children and providers and a high degree of cultural fit (Tonyan, 2017).

#### Colombia

In Colombia, the national law De Cero a Siempre (Ministerio de Educación Nacional, 2016) establishes commitment to access to quality early childhood care and education (Cosso et al., 2022). Public and private early childhood education services are delivered through center-based services (Centros de Desarollo Infantil- CDIs), as well as community-based and family-based programs targeting low-income and vulnerable children (Hogares Comunitarios de Bienestar-HCB) (Bernal et al., 2019). Previous research in Colombia suggests that early childhood centers have low to moderate quality, with limited learning materials, and varying levels of pedagogical quality of language, math, and science activities (Maldonado-Carreño et al., 2022). It also suggests that long-term exposure to the HCB program is associated with positive effects on cognitive and social–emotional development. However, several implementation issues, including inadequate portion sizes for children in the dietary protocol, have also been identified (Bernal et al., 2009). Colombia has national ECCE quality guidelines for both CDIs and the HCB program (Instituto Colombiano de Bienestar Familiar, 2019; Ministerio de Educación Nacional, 2014). In Barranquilla, Atlántico, where the present study took place, data were collected in consultation with the Atlántico Secretary of Education, as the study aligned with broader departmental efforts to improve ECCE quality.

#### Liberia

In Liberia, there are four types of facilities that provide early education: government; private; community-based; and faith-based. Most settings are designed for children ages three to six years, leading up to enrollment in primary schools (Republic of Liberia, 2011) Overage children, or those who are enrolling in school for the first time over the age of six years and up to 12 years, are present in every type of early childhood setting (Ministry of Education, 2016). There has been recent expanded access to ECCE, but quality of instruction remains low as the Liberian ECCE system faces severe resource constraints (Lipcan et al., 2018). There are national early learning standards in place (Ministry of Education, 2020). However, at present, the government does not regulate all early childhood settings regularly; in particular, private and faith-based schools are rarely regulated. Efforts to measure quality at scale are beginning and the data collected for this study were intended to generate a baseline of quality practices.

## Methodology

### Participants

Participants for this study were recruited in 2021 and 2022 in all settings in partnership with local organizations working in early childhood. As noted above, a mixed methods approach informed the study design and participants were engaged in both quantitative and qualitative data collection (Creswell, 2011). In Liberia and Colombia, recruitment was specifically done through training programs based at universities making accommodations for COVID-19. Participants included early childhood professionals in three settings as described below and summarized in Table 1.

**Table 1:** Description of participants, by country

	USA	Colombia	Liberia
Sample size	51	30	97
Profile of educators	Licensed family childcare providers	Public and private ECCE educators	Preschool educators
Average age of educators	37 years	37 years	34 years
Average number years of education	15 years (equivalent to an associate degree)	15 years (equivalent to a technical degree)	13 years (equivalent to a high school degree)

In Nebraska, licensed family childcare providers who were independent entrepreneurs caring for children in their homes were recruited through a partnership with a non-profit organization focused on childcare business coaching (*n* = 51) serving a large metropolitan area. In Nebraska, the average provider who participated in the study was 37 years old, female, and had an associate’s degree or Child Development Associate certificate (equivalent to 15 years of education starting from grade 1).

In Colombia, public and private ECCE programs (*n* = 30) were recruited in the Atlántico Department from a list from the Colombian Secretary of Education and the Colombian Institute of Family Welfare *(Instituto Colombiano de Bienestar Familiar: ICBF)* obtained by our research partner in the Department of Education at the Universidad del Norte. The data was collected in Malambo, Soledad, and Barranquilla. These three cities, which are located on Colombia's northern coast, offered a heterogeneous range of early childhood education school services. Barranquilla is the fourth most populated city in Colombia, and its economy is responsible for 18% of the value-added production in the Caribbean region. The average Colombian early childhood professional in the study was 37 years old, female, with a technical degree (equivalent to 15 years of education starting from grade 1. Participants were selected based on two inclusion criteria: first, they were required to be working in public or private childcare centers in Colombia that complied with national guidelines for the *De Cero a Siempre* early childhood education program (From Zero to Forever), and second, the selected early childhood programs were required to be accessible to researchers in the COVID-19 public health emergency context.

In Liberia, data was collected in two phases. During the first phase (*n* = 38), preschools were identified in three cities (Monrovia, Gbarnga, and Buchanan) through the Ministry of Education, Education Management Information System (EMIS) database. These cities were selected due the high level of support they received from local and international partners, on the grounds that examining quality in the most supported cities would also lend insight into quality in the areas with less support. During the second phase (*n* = 59), three out of the fifteen counties (Bong, Montserrado, and Grand Gedeh) were randomly selected from the Ministry of Education, Education Management Information System (EMIS) data based to represent the three educational regions (South East, North, and Central) of Liberia. Two districts were then selected from each participating county (one urban and one rural). In each district, ten preschools were selected to participate. Preschools were recruited by our research partner at the Teachers’ College at the University of Liberia to participate in the project with the support of the Ministry of Education. The average early childhood professional who participated in the study was 34 years old, with a high school degree (equivalent to 13 years of education starting from grade 1).

On average, early childhood professionals in Nebraska and Colombia were of similar age (37 years old) with similar education levels (15 years), while early childhood professionals in Liberia were slightly younger (34 years) with less years of education (13 years).

### Measures

The Brief Early Childhood Quality Inventory (BEQI) was developed to easily index evidence-based practices in early childhood settings across contexts. The BEQI is comprised of key practices in ECCE, based on two related criteria: first, evidence that use of that practice has been associated with child outcomes; and second, the ability to define and measure the practice in a way that observers across countries would be able to observe easily and reliably. The BEQI was designed to be adapted to settings, with emphasis on clearly identifying aspects of early childhood environments that can be indexed with a yes/no format, to facilitate reliable data collection and ability to interpret the data. The content of the scale was generated using multiple sources of information.

First, items were identified on the basis of the author’s experience with cross-cultural quality measurement, namely the development, adaptation, and implementation of quality measurement using the MELE. The content of MELE was developed through extensive consultation with experts across countries (UNESCO, 2017) and was tested in several countries. Experience testing the MELE scales informed several goals of BEQI. These goals included: reducing the complexity of the scale by transforming the rubric from a 4-point scale to yes/no response options; adding more items focused on early childhood professionals’ practices to promote children’s social/emotional development; and adding items specifically focused on documenting children’s autonomy and choice in activities vs. the time spent in educator-directed activities.

Second, items were reviewed with early childhood professionals in all three countries and items were deleted, changed, or added to align to local priorities for quality. In Nebraska, a member of the research team with expertise in family childcare settings and quality indicators reviewed and revised the items. Next, the revised items were reviewed in collaboration with a local early childhood network who engages in quality coaching with family childcare providers, and with a group of family childcare providers directly, through focus groups and interviews with childcare professionals including visits to home childcare settings for individual feedback and group discussions with teams of childcare providers. In Colombia, the tools were translated and aligned to national ECCE quality guidelines from both the National Ministry of Education and Colombian Family Welfare Institute. Then, ECCE experts from the Atlántico region’s Secretary of Education, Colombian Family Welfare Institute, ECCE centers, and teacher trainers reviewed the tools to ensure they were culturally appropriate and relevant to local priorities for ECCE quality. In Liberia, the national Ministry of Education’s Bureau of Early Childhood Education and Bureau of Teacher Education contributed to the review and adaptation process. The tools were aligned to the Liberian Ministry of Education’s Early Learning and Development Standards and priority areas identified by the Ministry of Education and for use by the teacher education and training program at the University of Liberia. The final list of items varied by country. A summary of items that were consistent across countries appears in Table 2.

**Table 2:** Frequencies of BEQI items, by country

Country	BEQI Item									
	Child choice	Children read books	Free play	Imaginary play	Gross motor activities	Music Activities	Engage with electronics	Go outside	Interact with peers	Peers mostly positive
Colombia (*N* = 30)	23%	10%	33%	43%	63%	80%	47%	17%	47%	93%
Liberia (*N* = 97)	0%	2%	0%	0%	14%	38%	3%	9%	40%	40%
Nebraska (*N* = 51)	100%	40%	94%	84%	84%	65%	26%	51%	100%	100%

Country	BEQI Item									
	Children work: alone	Children work: pairs	Children work: small groups	Children work: whole group	Children Work: 1:1	Balance structured free play^a^	Math activity	Math talk	Literacy activity	Literacy talk
Colombia	70%	7%	3%	100%	43%	13%	27%	27%	33%	0%
Liberia	55%	6%	4%	77%	18%	7%	59%	–^b^	57%	–
Nebraska	90%	80%	88%	80%	66%	32%	45%	84%	44%	86%

Country	BEQI Item									
	Science activity	Science talk	Comforts children	Interacts with children	Makes eye contact	Gets On children's level	Takes part in activities	Storybook read	Talk about feelings	Back and forth discussion
Colombia	23%	7%	61%	–	100%	20%	27%	13%	33%	57%
Liberia	19%	–	53%	–	81%	7%	12%	0%	24%	32%
Nebraska	36%	62%	98%	88%	–	–	80%	78%	49%	96%

Country	BEQI Item									
	Open-Ended questions in dialogue	Connect activities to daily experience	Close-ended questions	Rote instruction	Individualized instruction	Call children by name	Mostly positive	Does not use physical punishment	Intervene in peer conflicts	Misbehavior addressed
Colombia	57%	57%	100%	77%	37%	100%	93%	100%	70%	88%
Liberia	32%	22%	92%	77%	35%	87%	73%	68%	71%	79%
Nebraska	82%	60%	96%	59%	40%	92%	–	–	–	–

Country	BEQI Item									
	Do not use negative verbal interactions	Children are not left alone	Treat/ attend all children equally	Indoor space sufficient	materials organized by type	Materials representing diversity	Engage with educational toys^c^	Engage with writing materials^d^	Engage with art materials^e^	Engage with fantasy materials^f^
Colombia	27%	100%	100%	87%	97%	0%	27%	23%	73%	23%
Liberia	87%	61%	67%	15%	3%	–	0%	68%	9%	0%
Nebraska	94%	94%	86%	96%	98%	86%	–	–	–	–

Country	BEQI Item									
	Engage with Blocks^g^	At least 10 infant books	At least 10 preschool books	At least 10 school-aged books	Number of complete books^h^	Sufficient outdoor space	Outdoor gross motor equipment	Access to drinking Water	Soap and running water	Wash hands after toileting
Colombia	30%	–	–	–	30%	73%	77%	100%	87%	0%
Liberia	0%	–	–	–	3%	34%	14%	57%	26%	36%
Nebraska	–	71%	69%	49%	–	100%	98%	96%	–	88%

Country	BEQI Item									
	Access to sanitary toilet	Toilets separated by gender	Adequate light	Adequate ventilation	Free Of hazard	Secure building				
Colombia	80%	27%	90%	93%	27%	93%				
Liberia	57%	57%	51%	49%	60%	47%				
Nebraska	96%	–	100%	100%	98%	–				

^a^% of respondents with balance of free play and teacher planned. ^b^“–” Blank spaces indicate that the item was not included in that setting. ^c^% of respondents with children engaging with Educational Toys ^d^% of respondents with children engaging with Writing Materials. ^e^% of respondents with children engaging with Art Materials. ^f^% of respondents with children engaging with Fantasy Materials. ^g^% of respondents with children engaging with Blocks. ^h^% of respondents with 25 or more books. % of respondents with a balance of time spent in free play and teacher-planned activities

In each setting, recommendations for changing, adding, or deleting items were made based on the alignment of the items with country/context ECCE quality standards and the priorities for information on early childhood settings. For example, in Liberia, there was substantial interest in addressing the physical safety of settings and in assessing the use of physical punishment. In the USA, there was emphasis placed on whether children were read to daily, while items on the overall physical safety of the setting were considered less relevant. In addition to BEQI, early childhood professionals were given a brief survey of demographic characteristics including formal education, age, and years of experience in early childhood. Given country variation in education systems, the categories for education were based on each country’s educational system, and then were standardized by counting the years of formal education.

#### Training And Data Collection

Observers were trained to reliability on BEQI items in all three contexts. Training included a 12-h workshop to explain the structure of the scale and an in-depth review of items. Classroom videos were used to provide examples of each item in the context of local settings. Observers practiced scoring to obtain initial reliability during the workshop. The average agreement with master code with classroom videos was 86% in Liberia, 86% in Colombia, and 91% in the USA. In addition to passing video reliability quizzes, all observers were required to pass written quizzes on the items and BEQI data collection procedures; all achieved scores above 85%. In all three countries, observers then conducted 20% of observations in pairs while scoring independently and reliabilities were calculated based on percent agreement of items across observers. Because much of the data collection took place during COVID, workshops were held virtually in the USA, in-person in Liberia, and a combination of virtual and in-person in Colombia. Data collection in the USA took place virtually for all observations; in Liberia and Colombia, observers traveled to schools to complete in-person observations.

## Results

Our first step in analyses used qualitative approaches to generate a list of items for each setting, and then to use quantitative methods to describe the settings according to items selected for use within each context. The mix of items used to index each construct varied by setting. As seen in Table 2 above, results indicated that the frequencies of items varied by country.  Below we present narrative findings of selected results.

*Nebraska (n* = *51)*. ECCE settings in Nebraska were characterized by high levels of child choice and free play, with 100% of settings with child choice and 94% reporting free play. Less than half of the settings were observed with children engaging in reading on their own. Children frequently were observed engaging in imaginary play and gross motor activities (84% of observations). In all settings, children engaged with peers and those interactions were observed as positive. A little less than half of the settings had math, literacy, or science activities. Eighty percent of early childhood professionals engaged in play with children and 78% read a book. Back and forth discussion and open-ended questions were frequent, at more than 80% of settings. Connecting classroom activities to children’s lives was less frequent at 60% of settings. In all, ECCE settings were largely child-directed, with many opportunities for engagement with early childhood professionals and peers.

*Colombia (n* = *30)*. ECCE settings in Colombia were characterized by relatively lower degrees of child choice and free play, with 23% of classrooms with some degree of child choice and 33% with free play time. In 10% of classrooms, children were observed engaging with books on their own. Music was common with 80% of classrooms engaging in music while imaginary play was less frequent at 43% of observations. In about half of the classrooms, children engaged with peers. Math, literacy, and science activities took place in about 25–30% of classrooms. Less than 30% of early childhood professionals engaged in play with children whereas more than half connected classroom activities to children’s daily lives. In all, ECCE settings were somewhat child-directed, with some opportunities for children to engage in play and interact with peers.

*Liberia (n* = *97).* In Liberia, ECCE settings were characterized by high degrees of educator-directed activities; in 91% of classrooms observed, children spent most of their time in educator-planned activities. Children were not observed as having choice in how to carry out classroom activities or free play in any classrooms. In 2% of classrooms, children were observed reading or looking at books on their own. In 38% of classrooms, children engaged in music activities; in 14% of classrooms, children engaged in gross motor activities. In 40% of classrooms, children interacted with their peers and in 18%, children worked one-on-one with their educator. In more than half of classrooms children engaged in math activities (59%) and literacy activities (57%); while science activities were less commonly observed (19%). Early childhood professionals were observed engaging in play with children in 12% of classrooms. Seventy-seven percent of classrooms engaged in rote instruction. Back and forth discussion and open-ended questions were less frequent, observed in 32% of settings. In all, ECCE settings were mostly educator-directed, with almost no opportunities for child choice and some opportunities for children to interact with peers.

To address our second research question, whether any items showed initial signs of feasibility as anchor items across countries, we conducted analyses to assess the degree of correlation between items. By anchor item, we mean a set of common items used to facilitate the comparison of scores across countries (Angoff, 1984; de Ayala, 2009) that allows for score comparisons across settings. Using polychoric correlations, standards for identifying possible anchor items across countries included adequate variability within each country and similar bivariate associations with other items. The polychoric correlation is used whenever one is doing factor analysis with ordinal variables. If one were to use Pearson correlations for factor analysis with variables that have 5 categories or less, then factor loadings are known to be inaccurate (see, e.g., Bandalos, 2018) so the polychoric correlation is used and reported here. As displayed in Table 3, results indicated that a small set of items showed similar correlational properties across countries indicating their possible use as anchor items: literacy activities; science activities; use of rote instruction; negative verbal interactions between early childhood professionals and children; music activities; children working alone; children working in pairs; going outside; and gross motor activities. The small sample sizes precluded testing models across countries, but initial results indicated that a workable factor structure would likely be unifactorial rather than multi-dimensional, given the small number of items indicating possible use as anchor items across countries.

**Table 3:** Polychoric correlation matrix of the item response data in Nebraska (N), Colombia (C), and Liberia (L)

	(1)			(2)			(3)			(4)			(5)			(6)			(7)		
	N	C	L	N	C	L	N	C	L	N	C	L	N	C	L	N	C	L	N	C	L
Gross motor activities	1	1	1																		
Music activities	.25	.76	.61	1	1	1															
Go outside	.63	−.39	.53	−.11	−.40	−.07	1	1	1												
Children work: alone	−.97	−.60	−.14	−.98	−.29	−.09	−.43	−.18	−.46	1	1	1									
Children work: pairs	−.98	−.18	−.95	−.52	.96	.15	.04	.50	.19	.34	−.27	.34	1	1	1						
Children work: small groups	.02	.97	−.95	−.03	.94	−.99	.52	−.95	−.95	−.95	−.99	−.06	.25	.99	−.94	1	1	1			
Children work: 1:1	.46	−.48	−.06	−.42	−.41	−.28	.34	−.99	.09	.63	.99	.60	.10	−.98	.26	−.01	−.98	−.95	1	1	1
Math activity	.34	−.28	−.08	.02	−.45	.01	.16	−.99	.29	.38	.12	.15	.29	−.97	−.12	−.32	−.96	−.40	.75	.60	−.20
Literacy activity	.32	.41	.62	.12	.35	.40	.20	−.98	.31	.36	.00	.06	.09	.22	.12	.16	.98	.22	.45	.15	−.18
Science activity	−.03	−.12	.25	.21	.16	.11	.47	.85	−.97	.26	−.27	.17	.99	.38	.24	−.21	−.95	−.95	.29	−.29	.47
Takes part in activities	.56	.54	.43	.11	.23	.30	.04	−.14	−.04	.00	−.17	.19	−.01	.32	.09	.25	.99	−.94	.70	−.12	.16
Rote instruction	−.28	.12	−.29	.57	.22	−.13	−.04	−.32	.97	−.02	−.03	−.35	−.41	.96	−.16	−.12	.95	−.03	.30	.29	.28
Negative verbal interactions	−.95	.54	−.03	−.98	.23	.12	.98	−.14	−.39	−.99	.12	−.23	−.97	.32	.94	−.95	.99	−.21	−.03	−.12	.06

	(8)			(9)			(10)			(11)			(12)			(13)					
	N	C	L	N	C	L	N	C	L	N	C	L	N	C	L	N	C	L			
Math activity	1	1	1																		
Literacy activity	.68	−.49	−.30	1	1	1															
Science activity	.70	−.99	−.25	.63	−.99	−.32	1	1	1												
Takes part in Activities	.45	.25	.26	.46	.09	.15	.33	.04	.29	1	1	1									
Rote instruction	.43	−.04	.34	.36	.10	−.12	.31	.24	−.33	.48	−.34	.13	1	1	1						
Negative verbal interactions	−.28	−.38	−.60	−.98	.35	−.19	−.15	.04	.30	−.96	.25	.16	−.12	−.34	−.17	1	1	1			

## Discussion

Creating feasible and contextually relevant measures of quality can play an important role in scaling quality early childhood programs. Our results offer a new approach to measuring quality that begins with engagement of stakeholders to generate a list of relevant items and descriptions of typical ECCE environments using a simple checklist. This study contributes initial findings from this mixed methods approach with the potential to identify anchor items with relevance across settings. From this study, we offer several conclusions with implications for measurement of ECCE quality at scale.

First, we provide concrete and specific evidence that the range of practices varies considerably from one context to the next, with substantial variation in the number of children in each setting, the roles of early childhood professionals in shaping children’s experiences, from predominantly educator-led to predominantly child-led, and in the experiences of children within each setting in terms of the activities and dialogue with adults. For example, in the USA, every setting provided children with free choice but only a small portion engaged children in literacy or math activities; whereas in Liberia, no setting offered children free choice, but most engaged in math or literacy activities. The extent to which countries varied on these practices suggests that one scale is unlikely to capture meaningful variation from one setting to the next within each context. More centrally, the significance of these differences for child development in each context is not yet fully understood and requires local research with each population to fully define aspects of quality that are both culturally grounded and significant for child development.

Second, consistent with the idea that there are potentially some paths toward cross-cultural measurement, our work identifies a set of items that could serve as anchor items across settings. Our list included a range of activities as well as some aspects of educator/child interactions, including early childhood professionals’ use of rote instruction and negative interactions with children. While these items represent only a small set of possible quality constructs, when taken as a set of indicators, these items show promise as a first step in creating a core set to adapt and expand in different settings. Because existing work has largely failed to replicate more complex factor structures and scales from one place to the next (Betancur, 2021), an alternative is to rely on a smaller, more discrete set of items rather than large, complex scales when initiating quality measurement in diverse settings. The BEQI items could serve as a starting point for more extensive adaptation and addition of items that correspond to specific quality goals. Identifying an anchor set of items would help satisfy a global need for a feasible measure of quality that can be used at scale and responds to recent calls for more evidence on the extent to which children experience nurturing environments globally during the preschool years (McCoy et al., 2022).

There are several limitations to our study. The conclusions we draw here are based on small samples from settings that were intentionally diverse from one another, which may have led to large differences in quality practices that are specific to this study. Thus, the set of anchor items we identified may similarly be specific to this study. The narrow range of items that emerged as workable across settings may be a function of the limited number of items that were deemed relevant across settings during the adaptation process, although we maintain that the selection of items is an important indicator of goals for quality within each context. We also do not have extensive measures of educator/child interaction, which is a shortcoming given the importance of relationships for children’s development (Bergin et al., 2009; Mashburn et al., 2008).

Finally, an important next step for our work is to document associations between BEQI and child development within each context. The question of whether quality measures are related to child development is a central concern as programs scale, not only for research but for implementation of high-quality programs (Burchinal, 2018; Yoshikawa et al., 2018). In many countries, measurement of some kind is integrated into large-scale assessments of the quality of early childhood systems through monitoring and quality assurance (Yoshikawa, et al., 2018; Author citation redacted). Existing work on quality measurement in diverse contexts, often measured using adaptations of measures originally developed for high-income countries, has generally relied on small samples and/or controlled trials (Moore et al., 2008; Aboud et al., 2011). In cases where measurement has been used across large populations, results linking existing quality measures to child outcomes tend to show mixed to small effects (Raikes et al., 2020; Wolf & McCoy, 2019). The significance of these small effects is twofold: first, the variation in practices from one setting to the next may require measures that are attuned to context; and second, the most meaningful variation for countries to capture may be between settings within the same context. However, it is equally important to note that research to date suggests that children benefit from stimulating and supportive environments (Britto et al., 2017) and indeed, there are associations of quality ECCE with children’s development across countries (for example, see Rao et al., (2017) on the impacts of ECCE across countries; see McCoy et al. (2018) and Wolf et al., (2019) on aspects of quality associated with child outcomes in Ghana; von Suchodoletz et al., under review). The question, then, is clarifying which aspects of quality are most important in each setting, and creating measures that generate useful information for improvement while avoiding floor and ceiling effects. However, we do not yet know whether current definitions of quality, even those that are promoted by governments through quality standards, are in fact relevant to all early childhood settings.

It is also possible to conclude from this study that identifying one measure or set of items to work across contexts is inappropriate given the range of practices that characterize each setting and the lack of evidence on which practices are most critical for child development. We also must be mindful of the potential drawbacks of defining quality too narrowly and thus overlooking or discouraging practices that are in fact beneficial to children’s development in specific contexts or cultures. For example, some theorists have argued that quality should be defined by the alignment of children’s opportunities for learning with local values and priorities (Tonyan, 2017). This definition maintains the priority on providing children with opportunities for growth and stimulation while integrating the critical notion of cultural and contextual priorities.

Decades of empirical and theoretical work have established that child development is influenced by the quality of environmental inputs–and that some aspects of human development, such as the role of environmental stimulation and support in encouraging learning–are universal, albeit manifested differently in different settings. While the clarification of universal vs. culturally specific elements of quality in early childhood settings may require extensive research to untangle, we believe that addressing these questions is both theoretically important and has practical significance, as it can serve as the basis for globally relevant measures of quality. At the same time, while several studies have reported on adaptations of scales, the substantial diversity in goals and practices suggests that the adaptation required may be so extensive that the original assumptions of the scales are called into question. As well, there has been little work focused specifically on the degree to which early childhood professionals, stakeholders, and government officials can use the information that comes from quality measures to make improvements to practice. Taken together, our results suggest that using an approach to quality measurement that intentionally takes into account the need for local adaptation, while still based on evidence-based practices that promote children’s development, is one path forward that allows for greater localization of quality results.

Our results also have implications for ECCE policy, specifically the integration of data into ECCE systems in ways that promote improvement. Overall, we maintain that the most powerful purpose of quality measurement may be to provide useful and relevant feedback to early childhood systems, especially as programs are scaled nationally. Further, although more evidence is needed, existing evidence suggests that actionable, relevant data comes from measures that are perceived to be aligned with local context. For maximum impact, measures should be designed to be feasible and simple, with ample room to adjust the content of scales to match the settings in which they are used (Farran et al., 2017), however many existing quality tools are complex and difficult to translate into consistent changes in practice (Hanno, 2022). Focusing on descriptive information that provides clear directions for professional development may be more useful than using scales or summary scores that integrate multiple elements and thus may be more susceptible to cultural assumptions about appropriate care.

Tools such as BEQI can offer a new path forward as a ground-up approach to measurement, in which local goals for quality and needs for locally relevant data drive decisions on what to include on quality measures, starting with a list generated through available scientific and empirical evidence on child development. These types of monitoring tools may have the potential to respond to the need for effective quality assurance systems in both high-income (Burchinal, 2018; Farran et al., 2017) and low- and middle-income countries (Yoshikawa, et al., 2018). As early childhood programs are scaled, emphasis on effective, culturally, and contextual relevant measurement tools is an essential piece of achieving the vision of investment in early childhood as a pathway toward equity.
